# First-Principles Study of Biaxial Strain Effects on Schottky Barrier Modulation in Graphene/ZnSe Heterostructures

**DOI:** 10.3390/nano15231816

**Published:** 2025-12-01

**Authors:** Guowang Pang, Xue Wen, Lili Zhang, Yineng Huang

**Affiliations:** 1 College of Science, Xinjiang Institute of Technology, Aksu 843100, China; gwpang@xjit.edu.cn; 2 Xinjiang Laboratory of Phase Transitions and Microstructures in Condensed Matters, College of Physical Science and Technology, Yili Normal University, Yining 835000, China; 19899036782wx@sina.com; 3 National Laboratory of Solid State Microstructures, College of Physics, Nanjing University, Nanjing 210093, China

**Keywords:** Schottky barrier, heterostructure, first principles, graphene/ZnSe

## Abstract

Reducing the Schottky barrier at the metal–semiconductor interface and achieving Ohmic contact is crucial for the development of high-performance Schottky field-effect transistors. This paper investigates the stability, interface interactions, interlayer charge transfer, and types of Schottky contacts in the graphene/ZnSe heterostructure structure using first-principles methods. It employs biaxial strain as a control mechanism. The results indicate that applying compressive strain increases the barrier and band gap while maintaining n-type contact; whereas tensile strain reduces the n-type barrier to negative values, inducing Ohmic contact and decreasing the band gap. The findings of this study will provide theoretical references for the design and fabrication of field-effect transistors, photodetectors, and other optoelectronic devices.

## 1. Introduction

In recent years, the development of traditional silicon-based semiconductor devices has been gradually approaching its physical limits [1]. As the transistor channel length scales down to the nanoscale, effects such as quantum tunneling have become increasingly prominent, leading to significant performance degradation and posing severe challenges to the further miniaturization and performance enhancement of integrated circuits [2,3]. Against this backdrop, electronic devices based on novel low-dimensional materials have emerged as a crucial direction for overcoming these existing technological bottlenecks.

ZnSe is a direct bandgap semiconductor material [4] with a bandgap value of 2.7 eV at room temperature [5]. It possesses advantages such as a wide bandgap, direct transitions, and high temperature resistance. Its excellent optoelectronic properties have led to widespread applications in optics, sensors, and optoelectronic materials [6,7]. As a member of the two-dimensional transition metal dichalcogenide family, the bandgap of 2D ZnSe can be tuned through methods such as adsorption, doping, external electric fields, functionalization, and patterned design [8]. Furthermore, studies have shown that ZnSe(C_2_H_8_N_2_)_1_/_2_ exhibits high electron mobility, making it highly suitable for optoelectronic devices and field-effect transistors (FETs) [9]. Constructing a heterostructure by combining 2D ZnSe with graphene via weak van der Waals interactions can not only preserve the inherent advantages of each 2D material, but also introduce novel physical phenomena, thereby compensating for the zero-bandgap limitation of graphene [10]. This approach provides new pathways for research in fields such as FETs [11], photodetectors [12], and integrated circuits [13]. Currently, numerous graphene-based heterostructures, such as graphene/ZnO [14], graphene/h-BN [15], graphene/g-GaN [16], graphene/GaSe [17], and graphene/MoS_2_ [18] have been extensively studied. Forming graphene-based heterostructures can open a bandgap at the Dirac point of monolayer graphene and create a gap of a certain width. Concurrently, the absence of Fermi-level pinning in graphene-based FETs facilitates the formation of low-contact resistance [19]. Additionally, research has found that applying biaxial strain is an effective method for modulating the interface properties and Schottky contact type of heterostructures. For instance, Qian et al. [20] applied biaxial strain to a MoSH/WSi_2_N_4_ Schottky junction and observed a gradual decrease in the Schottky barrier height under ±8% strain, accompanied by a transition from p-type to n-type contact. Ruixia Miao et al. [21] investigated the tuning mechanism of a 2D Janus Ga_2_SSe/β-Ga_2_O_3_ heterostructure under biaxial strain, revealing significant effects on the bandgap type, carrier effective mass, and optical absorption properties. Duoqiao Pan et al. [22] demonstrated that biaxial strain effectively modulates the electronic structure and optical properties of a g-ZnO/WS_2_ heterostructure. While the successful experimental synthesis of a graphene/ZnSe heterostructure has been reported [23], the microscopic mechanism of interfacial charge transfer in the graphene/ZnSe heterostructure and theoretical studies on the modulation of its properties by external fields warrant further in-depth investigation.

To expand the application scope of monolayer graphene and ZnSe, particularly for practical applications in nanodevices, this study employs first-principle calculations to investigate the effects of biaxial strain on the graphene/ZnSe heterostructure. Specifically, we examine how strain modulates the interfacial interactions, electronic structure, Schottky barrier height, and contact type transition. Furthermore, the underlying microscopic mechanisms are explored to provide valuable insights for the fabrication of field-effect transistors and photodetectors.

## 2. Materials and Methods

### Models and Parameters

All calculations based on density functional theory (DFT) were performed using the Vienna Ab initio Simulation Package (VASP (version 6.0)) [24]. The electron exchange–correlation interactions were described by the Perdew–Burke–Ernzerhof (PBE) functional within the generalized gradient approximation (GGA). To accurately account for van der Waals forces within the heterostructure system, the Tkatchenko–Scheffler (TS) dispersion correction was incorporated into the DFT calculations [25]. A plane-wave kinetic energy cutoff of 550 eV was adopted. A 3 × 3 × 1 ZnSe supercell and a 5 × 5 × 1 graphene supercell were constructed for structural optimization and electronic property calculations. The Brillouin zone was sampled using a 2 × 2 × 1 k-point mesh generated by the Monkhorst–Pack method [26] for the primitive cells. For the structural optimization of the graphene/ZnSe heterostructure, a 4 × 4 × 2 k-point mesh and a cutoff energy of 550 eV were employed. The convergence criteria for the electronic self-consistent iteration and ionic relaxation were set to 1 × 10^−5^ eV per atom for the total energy and 0.01 eV/Å for the force on each atom, respectively.

**Figure 1:**
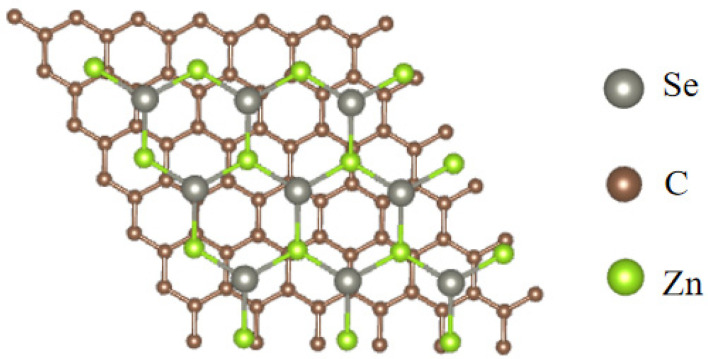
Graphene/ZnSe heterostructure model.

The initial lattice constants of monolayer ZnSe and graphene were a = 3.980 Å and a = 2.470 Å, respectively. After structural optimization, these values became a = 4.085 Å for ZnSe and a = 2.459 Å for graphene. The heterostructure model was constructed by stacking a 3 × 3 × 1 monolayer ZnSe supercell onto a 5 × 5 × 1 graphene supercell. The optimized heterostructure exhibits lattice constants of a = b = 12.332 Å, as depicted in Figure 1. A vacuum layer of 15 Å was introduced along the z-axis to eliminate any spurious interactions between periodic images.

## 3. Results and Discussion

### 3.1. Structure and Stability

To assess the structural stability, the binding energy was calculated as a function of the interlayer distance during the structural relaxation process. The binding energy was evaluated using the following formula:

(1) $$E_{coh}=E_{\left(ZnSe/graphene\right)}-E_{\left(ZnSe\right)}-E_{\left(graphene\right)}$$

**Figure 2:**
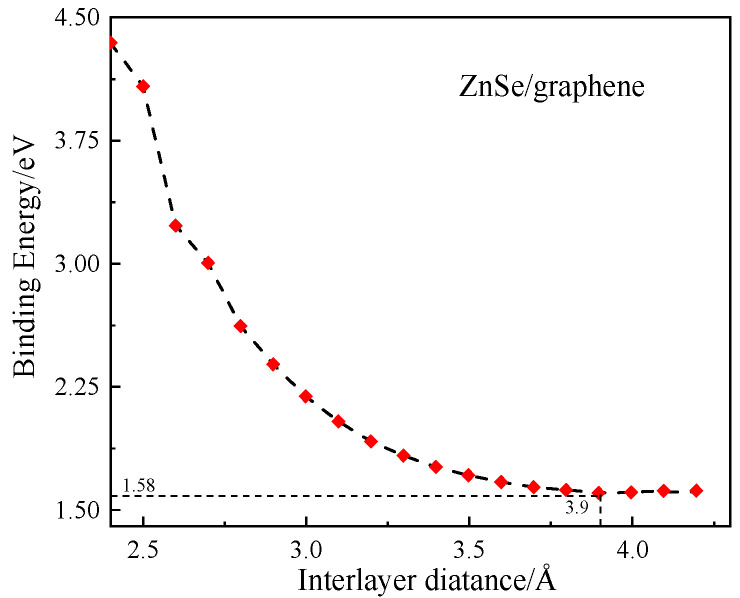
Relationship between binding energy of graphene/ZnSe heterostructure and layer spacing.

In the formula, $E_{\left(ZnSe/graphene\right)}$ represents the total energy of the optimized heterostructure, while $E_{\left(ZnSe\right)}$ and $E_{\left(graphene\right)}$ denote the total energies of the isolated, optimized monolayer ZnSe and graphene, respectively. A lower binding energy indicates greater structural stability. As shown in Figure 2, the binding energy reaches its minimum at an interlayer distance of d = 3.90 Å. Therefore, the most stable configuration of the graphene/ZnSe heterostructure corresponds to an interlayer spacing of 3.90 Å.

(2) $$\sigma =(a_{2}-a_{1})/a_{1}$$

The lattice mismatch rate of the graphene/ZnSe heterostructure was calculated using Formula (2) [27] to evaluate its lattice matching degree. In the formula, $a_{1}$ and $a_{2}$ represent the lattice constants of graphene and ZnSe, respectively. The calculated lattice mismatch is −3.319%, which is less than 5%, thereby satisfying the condition for a fully coherent interface [28]. The formation of the heterostructure will introduce lattice misfit energy. A more stable heterostructure system generally possesses a lower lattice misfit energy.

(3) $$∆E_{mismatch}=E_{(G)a^{\prime }}+E_{(ZnSe)a^{\prime }}-E_{\left(G\right)a}-E_{(ZnSe)a}$$

The lattice mismatch energy of the heterostructure was calculated using Equation (3) [29], where $E_{(G)a^{\prime }}$ and $E_{(ZnSe)a^{\prime }}$ represent the total energies of graphene and ZnSe after lattice optimization, while $E_{\left(G\right)a}$ and $E_{(ZnSe)a}$ denote the total energies of the optimized graphene and ZnSe systems, respectively. The calculated lattice mismatch energy of the heterostructure is 1.548 eV/nm^2^. This relatively small value prevents significant lattice strain, ensuring that the computational results align more closely with actual experimental conditions. To confirm that the interlayer interaction in the heterostructure is governed by van der Waals forces [30], the strength of these interactions was evaluated using Equation (4), where $\left|∆E_{mismatch}\right|$ is the lattice mismatch energy and $\left|E_{coh}\right|$ is the binding energy. The calculated van der Waals interaction energy for the graphene/ZnSe heterostructure is 3.128 eV/nm^2^, which is consistent with previously reported results in the literature [31].

(4) $$∆E_{vdw}=\left|∆E_{mismatch}\right|+\left|E_{coh}\right|$$

**Figure 3:**
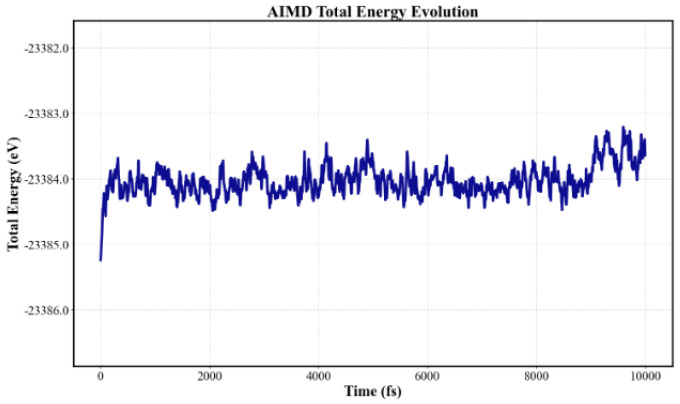
Total energy and temperature AIMD curve of graphene/ZnSe heterostructure system.

To further evaluate the stability of the graphene/ZnSe heterostructure, ab initio molecular dynamics (AIMD) simulations were employed to assess its thermodynamic stability [32]. As shown in Figure 3, at a temperature of 300 K, the system remains stable after 10,000 simulation steps with a 1 fs time step. Although slight structural fluctuations are observed, the energy variations are minimal, and the overall structure remains intact. These results confirm that the heterostructure exhibits good thermodynamic stability at 300 K.

### 3.2. Electronic Structure of the Heterostructure

**Figure 4:**
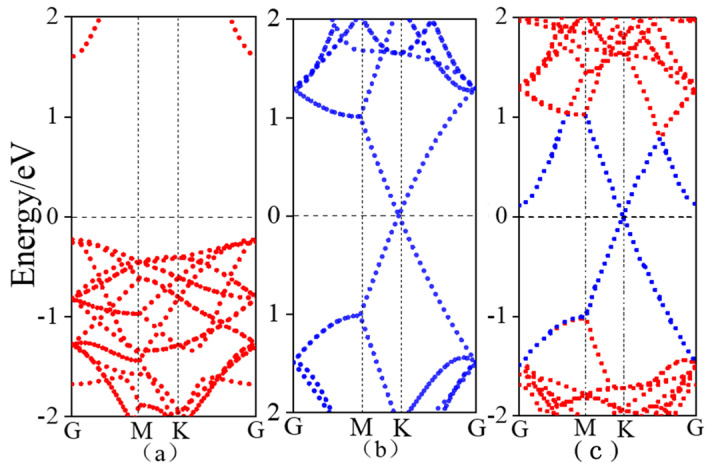
Band structure diagram (**a**) monolayer ZnSe; (**b**) graphene; (**c**) graphene/ZnSe.

Figure 4 displays the band structures of monolayer ZnSe, graphene, and the graphene/ZnSe heterostructure. As shown in Figure 4a, monolayer ZnSe is a direct bandgap semiconductor with a bandgap of 1.76 eV, which agrees well with previously reported computational results [33,34]. Figure 4b presents the band structure of graphene, where the conduction band minimum and valence band maximum meet at the Fermi level at the high-symmetry K point, resulting in a zero bandgap and characteristic metallic behavior [35]. Figure 4c illustrates the band structure of the graphene/ZnSe heterostructure. Compared to the individual components, the heterostructure’s band structure can largely be viewed as a superposition of the bands of monolayer ZnSe and graphene, well preserving their original electronic features. This suggests that the interlayer interaction is primarily governed by van der Waals forces, consistent with the computational findings discussed earlier. In the graphene/ZnSe heterostructure, the Dirac cone remains clearly visible, while the conduction band of ZnSe shifts downward as a whole. This phenomenon is related to the interlayer charge distribution: a potential difference between graphene and monolayer ZnSe leads to charge transfer from graphene to ZnSe at the interface. According to the Schottky–Mott model [36], the energy differences between the band edges and the Fermi level can be described by the n-type Schottky barrier height (n-SBH) and p-type Schottky barrier height (p-SBH). As indicated in Figure 4c, the conduction band minimum is closer to the Fermi level than the valence band maximum, with an n-SBH of 0.11 eV, which is smaller than the p-SBH of 0.98 eV. This confirms the formation of an n-type Schottky contact between ZnSe and graphene. Our calculation results are consistent with the reported n-type Schottky contacts in graphene-based heterostructures, such as graphene/MoSe_2_ [37] and WS_2_/graphene [38].

### 3.3. Three-Dimensional Charge Density Difference

**Figure 5:**
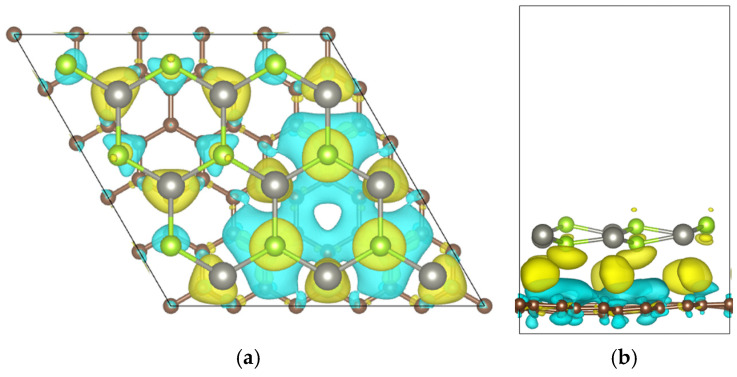
Charge density difference of the Graphene/ZnSe heterostructure: (**a**) top view and (**b**) side view. (isosurface = 0.0002 e/Bohr^3^. The color yellow represents the charge increase and the color cyan represents the charge decrease).

To investigate the charge transfer at the graphene/ZnSe heterointerface, the charge density difference was analyzed. As shown in Figure 5, yellow and cyan regions represent charge accumulation and depletion, respectively. The analysis reveals that charge is transferred from the graphene interface to the ZnSe layer. The carbon atoms in graphene donate a total charge of 0.0723 e. This leads to electron accumulation on the ZnSe side, causing a shift of its Fermi level toward lower energies, which is consistent with the band structure analysis. Concurrently, the loss of electrons increases the hole concentration in graphene, while the gain of electrons raises the electron concentration in the ZnSe layer. These effects collectively induce a built-in electric field at the interface directed from graphene to ZnSe, in agreement with the band analysis.

The band edge potentials of semiconductors can be estimated using the average electronegativity method [39].

(5) $$E_{VB}=\chi -E_{e}+0.5E_{g},\;E_{CB}=E_{VB}-E_{g}$$

**Figure 6:**
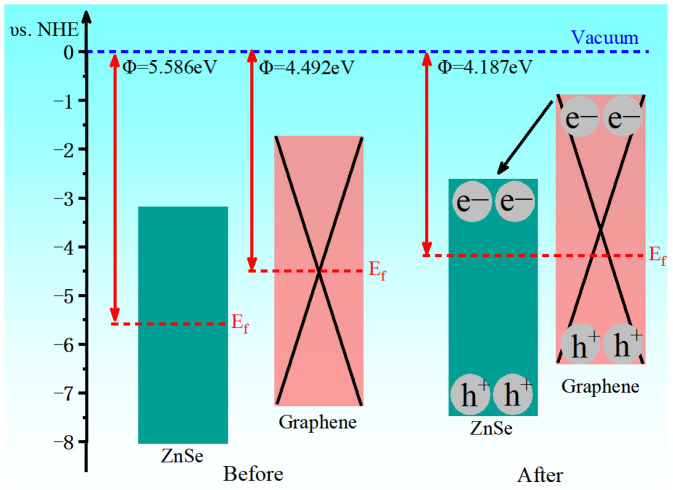
Band edge position diagram before and after contact between ZnSe and graphene.

In the above equation, *χ* represents the absolute electronegativity of the multicomponent semiconductor, *E_e_* is the energy of free electrons on the hydrogen energy scale, and *E_g_* denotes the band gap of the semiconductor. Figure 6 illustrates the variation in the band edge positions of ZnSe before and after contact with graphene. Upon heterostructure formation, the band edges of ZnSe shift as a result of Fermi level alignment between the two materials. The work function of the graphene/ZnSe heterostructure is determined to be 4.817 eV, which lies between those of pristine ZnSe and graphene. This observation can be attributed to interfacial charge transfer between the two components.

### 3.4. Modulation of the Schottky Barrier in Graphene/ZnSe Heterostructures by Biaxial Strain

**Figure 7:**
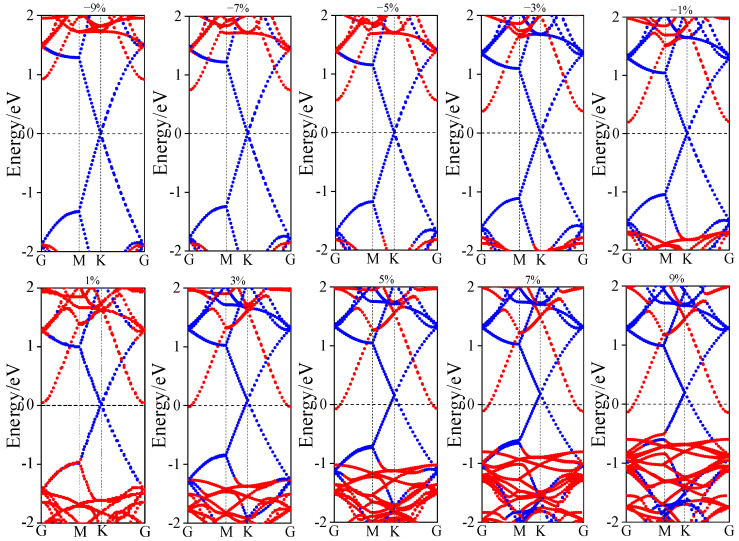
Band structure of graphene/ZnSe under different biaxial strains (−9~+9%).

Strain engineering serves as a viable technique for tuning the electronic band structure of semiconductor materials and their heterostructures. Figure 7 illustrates the electronic structure evolution under applied strain. Under compressive strains of −1%, −3%, −5%, −7%, and −9%, both the conduction band minimum (CBM) and valence band maximum (VBM) shift away from the Fermi level with increasing strain magnitude. This results in an increase of the n-type Schottky barrier height (SBH) from 0.172 eV to 0.898 eV. Throughout this range, the Fermi level remains closer to the CBM than to the VBM, indicating that the n-type SBH is consistently lower than the p-type SBH and the heterostructure maintains an n-type Schottky contact. In contrast, under tensile strains of +1%, +3%, +5%, +7%, and +9%, the VBM moves closer to the Fermi level while the CBM crosses it. The n-type SBH progressively decreases to zero, and the p-type SBH is reduced from 0.952 eV to 0.489 eV. The n-type SBH remains lower than its p-type counterpart under all tensile strains. These results demonstrate that the heterostructure transitions from an n-type Schottky contact to an ohmic contact with increasing tensile strain. Furthermore, examining the band gap evolution of the ZnSe layer reveals that compressive strain widens the band gap, whereas tensile strain significantly reduces it. Therefore, the application of tensile strain effectively lowers the Schottky barrier height, induces a transition in contact type, and narrows the band gap.

**Figure 8:**
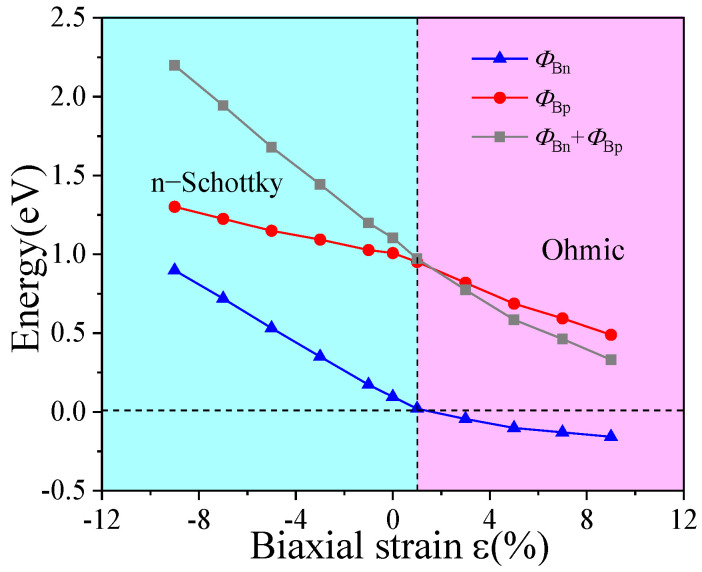
Variation curves of graphene/ZnSe heterostructure Schottky barrier heights under different biaxial strains.

A quantitative analysis based on the Schottky–Mott model was also conducted here, and the variation of Schottky barrier heights under different tensile and compressive strains is plotted in Figure 8. As observed, the Schottky barrier heights generally increase under compressive strain. The n-type Schottky barrier heights (n-SBHs) are 0.172 eV, 0.351 eV, 0.531 eV, 0.719 eV, and 0.898 eV, showing a significant increase with the intensity of compression. In comparison, the p-type Schottky barrier heights (p-SBHs) are 1.027 eV, 1.093 eV, 1.149 eV, 1.225 eV, and 1.301 eV, exhibiting a more gradual upward trend. Although both types of barriers rise with increasing compressive strain, the n-SBH remains consistently lower than the p-SBH. Therefore, the heterostructure retains its n-type Schottky contact character under compressive strain, consistent with the unstrained case. In contrast, under tensile strain, the Schottky barrier heights overall decrease. The p-SBHs are measured as 0.952 eV, 0.819 eV, 0.687 eV, 0.593 eV, and 0.489 eV, gradually declining with increasing tensile strain. Notably, the n-SBH drops to zero under only 1% tensile strain and further becomes negative, indicating a transition from n-type Schottky contact to ohmic contact. Furthermore, when tensile strain is applied starting from a structure pre-compressed by −8%, the bandgap of the ZnSe layer continuously narrows with increasing tensile intensity. These results demonstrate that applying biaxial strain to the graphene/ZnSe heterostructure can effectively modulate both the Schottky barrier height and the contact type.

## 4. Conclusions

Based on first-principle calculations, this study systematically investigates the structural stability and electronic properties of the graphene/ZnSe heterostructure. The results demonstrate that the structure is stable and represents a typical van der Waals heterostructure. At the equilibrium interlayer spacing, the heterojunction exhibits an n-type Schottky contact. The application of biaxial strain enables effective modulation of the contact type: compressive strain increases the n-type Schottky barrier height from 0.172 eV to 0.898 eV and the p-type barrier height from 1.027 eV to 1.301 eV, while maintaining n-type contact characteristics; whereas tensile strain reduces the n-type barrier below zero, inducing a transition to ohmic contact and significantly narrowing the band gap. This research demonstrates that the graphene/ZnSe heterostructure possesses a stable configuration and tunable electronic properties, allowing for controllable modification of contact types and band gap behavior through external strain fields.

## Data Availability

The original contributions presented in this study are included in the article. Further inquiries can be directed to the corresponding authors.
